# Human papillomavirus viral load in predicting high-grade CIN in women with cervical smears showing only atypical squamous cells or low-grade squamous intraepithelial lesion

**DOI:** 10.1590/S1516-31802003000600004

**Published:** 2003-11-06

**Authors:** André Luis Ferreira Santos, Sophie Françoise Mauricette Derchain, Marcos Roberto Martins, Luís Otávio Zanatta Sarian, Edson Zangiacome Martinez, Kari Juhani Syrjänen

**Keywords:** Human papillomavirus, Viral load, Cervical intraepithelial neoplasia, Papilomavírus humano, Carga viral, Neoplasia intra-epitelial cervical

## Abstract

**CONTEXT:**

Human papillomavirus (HPV) viral load may have an important role in predicting high-grade cervical intraepithelial neoplasia (CIN) in women with cervical smears showing atypical squamous cells or LSIL.

**OBJECTIVE:**

To determine whether the assessment of the viral load of high-risk HPV DNA is useful in predicting the detection of high-grade cervical intraepithelial neoplasia (CIN2 and 3) in women referred because of cervical smears showing only atypical squamous cells or LSIL.

**TYPE OF STUDY:**

Cross-sectional

**SETTING:**

Colposcopy Clinic in a University hospital.

**METHODS:**

A series of 119 women referred because of atypical squamous cells or LSIL between August 2000 and April 2001 were included. All women were subjected to a new cervical smear, HPV testing for the high-risk types using hybrid capture II (HCII), viral load measurement in relative light units (RLU) and colposcopy, with cervical biopsies (n = 97). Cervical lesions were graded using the CIN classification.

**RESULTS:**

Cervical biopsies revealed CIN2 or CIN3 in 11% of the cases, equally among women referred because of atypical squamous cells or LSIL. The HCII test was positive in 16% of women with atypical squamous cells and 52% of those with LSIL (OR = 5.8; 95% CI 1.4 to 26.7). There was strong correlation between CIN2 or CIN3 and positivity for HPV DNA when this group was compared with women with only CIN1 or normal cervix (OR = 7.8; 95% CI 1.5 to 53.4). In ROC analysis for HCII in diagnosing CIN2 and CIN3, the area under the ROC curve was 0.784, and the viral load cutoff point of 10.0 RLU/cutoff presented 77% sensitivity and 73% specificity. Second cytology showing at least atypical squamous cells did not accurately detect CIN2 or CIN3 (OR = 6.4; 95% CI 1.0 to 50.9). The sensitivities of the second cervical smear and HCII were similar, although the specificity of HCII was significantly higher than the second cervical smear.

**CONCLUSIONS:**

The viral load of high-risk HPV types was significantly associated with the diagnosis of CIN2 or CIN3 in women referred because of atypical squamous cells and LSIL abnormalities in their cervical smear.

## RESUMO

**CONTEXTO:**

A determinação da carga viral do papilomavírus humano (HPV) pode ter importante papel na detecção de neoplasia intraepitelial cervical (NIC) de alto grau em mulheres com colpocitologia apresentando células escamosas atípicas ou sugestivas de lesão escamosa de baixo grau.

**OBJETIVO:**

Avaliar se a determinação da carga viral do DNA HPV é útil para predizer a detecção da neoplasia intra-epitelial de alto grau (NIC2 e 3) em mulheres referidas por colpocitologias mostrando apenas células escamosas atípicas ou lesão intra-epitelial de baixo grau.

**TIPO DE ESTUDO:**

Transversal

**LOCAL:**

Serviço de colposcopia de hospital universitário.

**MÉTODOS:**

Foram incluídas 119 mulheres encaminhadas por células escamosas atípicas e lesão intra-epitelial de baixo grau entre agosto de 2000 e abril de 2001. De todas as mulheres foi coletada nova colpocitologia, espécime para teste de HPV usando captura de híbridos II (CHII), carga viral medida em unidades relativas de luz (URL). Foi realizada colposcopia com biópsia cervical em 97 mulheres. As lesões cervicais foram classificadas usando a classificação NIC. Para diagnóstico final, a colposcopia normal ou a presença de cervicite confirmada por biópsia foram classificadas como colo normal. Para análise estatística foram calculados o odds ratio (OR), com intervalo de confiança em 95%, e foi traçada uma curva "receiver operator characteristic" (ROC).

**RESULTADOS:**

As biópsias cervicais mostraram NIC2 ou 3 em 11% dos casos, igualmente distribuídas entre as mulheres encaminhadas por causa da presença de células escamosas atípicas ou lesão intra-epitelial de baixo grau. A CHII foi positiva em 16% das mulheres com células escamosas atípicas e em 52% daquelas com lesão intra-epitelial de baixo grau (OR = 5,8; IC 95% 1,4 a 26,7). Entre as mulheres com CHII positiva, 7% tinham cérvice normal, 73% NIC 1 (OR = 6,3; IC 5% 1,8 a 23,8) e 20% tinham NIC2 ou 3 (OR = 33,0; IC 95% 4,2 a 347,8). Na análise da curva ROC para CH II, diagnosticando NIC2 e 3, a área sob a curva foi de 0,784 e o ponto de corte da carga viral de 10.0 URL mostrou sensibilidade de 77% e especificidade de 70%. A segunda colpocitologia mostrando ao menos células escamosas atípicas não apresentou boa performance na detecção NIC 2 ou 3 (OR = 6,4%; IC 95% 1,0 a 50,9).

**CONCLUSÕES:**

A carga viral do DNA-HPV de alto risco oncológico foi significativamente associada com o diagnóstico de NIC2 e 3 em mulheres encaminhadas por detecção de células escamosas atípicas e lesão intra-epitelial escamosa de baixo grau na colpocitologia.

## INTRODUCTION

The clinical significance and management practices adopted in women with atypical squamous cells (ASC) or low-grade squamous intraepithelial lesion (LSIL) have not been uniformly accepted.^1-8^ Repeated cervical smears, addition of tests to detect human papillomavirus (HPV) DNA or referral of the women for immediate colposcopy are some of the different options, although they have clearly different cost-effectiveness. The preliminary results of the ASCUS/LSIL Triage Study (ALTS),^1^ an American national prospective randomized trial based on 3,488 women with atypical squamous cells of undetermined significance (ASCUS) and 1,572 women with LSIL, showed that hybrid capture II (HCII) testing for high-risk HPV DNA was frequently positive (around 85%) in women with LSIL. This therefore demonstrated the limited usefulness of the HCII assay in the management of these cases, because the vast majority of these women would be referred for (unnecessary) colposcopy due to the positive HPV tests. Regarding the women with ASCUS smears, Solomon et al. (2001) concluded that HCII testing for high-risk HPV DNA types was positive in 50.6% and should be a viable option for the management of these women. HPV DNA had greater sensitivity and specificity in detecting CIN3 (cervical intraepithelial neoplasia grade 3, carcinoma in situ or severe dysplasia) or above, in comparison with a single additional cervical test showing ASCUS or above.^1^

These results are not uniformly accepted, however.^4,9^ Recently, the Bethesda system modified their ASCUS category to make it two-tier: ASC–US (of undetermined significance) and ASC-H (cannot exclude high-grade squamous intraepithelial lesion - HSIL).^10^

According to the ASCCP (American Society for Colposcopy and Cervical Pathology) 2001 Consensus Guidelines for the Management of Women with Cervical Cytological Abnormalities,^8^ women with ASC-US should be managed using a program of two repeated cytology tests, immediate colposcopy or HPV DNA testing for high-risk HPV types. Women with ASC-H, LSIL, HSIL and atypical glandular cells should be referred for immediate colposcopy evaluation, regardless of the result of HPV testing. However, HPV testing for routine clinical management of ASCUS cytological abnormalities is not yet warranted^9-11^ and continues to be an investigational tool. Furthermore, evaluation of the clinical effectiveness of HPV testing combined with cervical smears needs to be completed in developing countries.

The aim of the present study was to shed further light on the issue and investigate whether HPV testing and viral load quantification are clinically useful tools for women who present with ASC or LSIL cervical smears in our settings in Brazil.

## MATERIAL AND METHODS

### Patients

In this cross-sectional study, we analyzed a series of 119 women aged 16 to 63 (median of 31 years), referred for the Colposcopy Clinic due to an abnormal cervical smear consistent with ASC (n = 19) or LSIL (HPV-suggestive changes in 37 women and CIN1 in 63), between August 2000 and April 2001. Women were excluded from the study if: a) they had a previous history of CIN or cervical, vaginal or vulvar cancer; b) they were referred because of an HSIL cervical smear; c) they had immunosuppression; or d) they were pregnant. The Ethical Committee of the Medical School Hospital approved the study protocol, and all participants gave their written informed consent.

All women were offered a questionnaire asking for their key sociodemographic data, and a new conventional cervical smear was taken, using the Ayre spatula and endocervical brush. This was fixed in 95% ethanol and stained by means of the modified Papanicolaou method. A second specimen was obtained from the endocervix using a Dacron swab and placed in 1.0 ml of specimen transport medium (Digene Corporation) for DNA HPV testing using the HCII test.

All women were subjected to colposcopic examination, according to routine practice, and the results were classified according to the International Federation of Cervical Pathology and Colposcopy (IFCCP) classification.^12^ The cervix was considered colposcopically normal when all of the squamous columnar junction was shown, and abnormal when the cervix presented some area of acetowhite epithelium, mosaicism, punctuation, leukoplasia or abnormal vessels. Abnormal areas were classified as major or minor abnormalities. In 90 women, colposcopically targeted biopsies were taken from suspicious areas. In addition, for seven women with unsatisfactory colposcopy, a large loop excision of the transformation zone (LLETZ) was done using a diathermy procedure. Twenty-two women with entirely normal colposcopy and a totally visible squamous columnar junction had no biopsy taken, and they were considered as having a normal cervix.

### Cytology and histology

Referral cervical smears were available for review from all the patients. The final cytological diagnoses for both the referral and the second cervical smear were obtained using the Bethesda System (2002)^10^ and were classified as negative, ASC, LSIL or HSIL (the second cervical smear only), based on a consensus review by two pathologists. Cervical biopsies were fixed in 10% phosphate-buffered formalin, embedded in paraffin, and stained with hematoxylin and eosin (HE). Biopsies were analyzed according to the World Health Organization criteria^13^ and classified as negative, CIN1, CIN2 or CIN3. Women with biopsyconfirmed cervicitis and those with normal colposcopy were classified as having a normal cervix in this study.

### Hybrid capture

The specimens for HCII were tested for probe B (high-risk HPVs: types 16, 18, 31, 33, 35, 39, 45, 51, 52, 56, 58, 59 and 68), and the test was classified as positive when the relative light unit/cutoff (RLU/CO) ratio (RLU of specimen/mean RLU of two positive controls) was 1 pg/ml or greater. These RLU/CO ratios also provided an estimate of the amount of HPV DNA in the specimens, i.e. the viral load in the sample. The storage of the specimens and all reagents, as well as the conduct of the tests, took place at the Medical School Hospital Laboratory, following the manufacturer's instructions (*Digene Diagnostics Inc., USA*).

### Statistical analysis

Mean HPV viral loads with standard deviation (SD) were calculated in relation to cervical smears and histological results. For ethical reasons, invasive diagnostic biopsy was indicated only in cases of repeated positive cervical smears or abnormal colposcopy. Beggcorrected estimate values for the sensitivity and specificity of HCII (at standard 1.0 RLU/CO cutoffs) and second cervical smears were calculated.^14,15^ Receiver operating characteristic (ROC) analysis was used for testing the diagnostic performance of the HCII test at cutoffs of 1.0 RLU/CO, 2.0 RLU/CO, 5.0 RLU/CO, 10.0 RLU/CO, 50.0 RLU/CO, 100.0 RLU/CO and 500.0 RLU/CO, and above the cutoff point, in order to detect histologically-confirmed CIN2 or CIN3 lesions.

All statistical analyses were done using the SAS software, version 8.0. Odds ratios (OR) with 95% confidence interval (95% CI) were used for evaluating the association between the HPV test result, cervical smear result and disease status.

## RESULTS

Altogether, 23 women (19%) were found to have a normal cervix via colposcopy, 91 (76%) presented with minor abnormalities and 5 (4%) with major abnormalities. Of the women with colposcopically guided biopsy areas, 6 (5%) presented with cervicitis in the histological analysis, 78 (66%) showed CIN1 and 13 (11%) had CIN2 or CIN3 (data not shown). Considering the distribution of these results according to the first cervical smears, only CIN1 was significantly higher in women referred because of LSIL cervical smears (OR 4.14; 95% CI 1.25 to 13.9). CIN 2 or CIN3 were found in the same proportions in women with ASC or LSIL (Table 1).

**Table 1:** Histological diagnosis and first cervical smear result in 119 women referred for colposcopy

Final disease status	First cervical smear		OR (95% CI)
	ASC (%)	LSIL (%)	
Normal cervix	9 (48%)	19 (19%)	REF
CIN 1	8 (42%)	70 (70%)	4.14 (1.25 to 13.9)
CIN 2 or 3	2 (10%)	11 (11%)	2.1 (0.4 to 21.2)
**Total**	**19 (100%)**	**100 (100%)**	

*CIN = cervical intraepithelial neoplasia; ASC = atypical squamous cells, LSIL = low-grade squamous intraepithelial lesion; OR = odds ratio; REF= reference values for odds ratios; CI = confidence interval.*

The HCII test was positive for the highrisk HPV DNA in 16% of the women (3 women) who had a cervical smear with ASC and 52% of those with LSIL. The HPV DNA detection rate was significantly higher (OR 5.8; 95% CI 1.4 to 26.7) in women with cytological changes consistent with LSIL than in those with ASC (Table 2).

**Table 2:** HPV DNA detection according to first cervical smear result in 119 women referred for colposcopy

Hybrid Capture II (1.0 RLU/CO)	First cervical smear		OR (95% CI)
	ASC (%)	LSIL (%)	
Negative	16 (84%)	48 (48%)	
Positive	3 (16%)	52 (52%)	5.8 (1.4 to 26.7)
**Total**	**19**	**100**	

*ASC = atypical squamous cells; LSIL = low-grade squamous intraepithelial lesion; OR = odds ratio; CI= confidence interval.*

The HCII and histology results are summarized in Table 3. It is noteworthy that 85% of the women with CIN2 or CIN3 had a positive HPV DNA test with a 1.0 RLU/CO cutoff point. There was a strong correlation between CIN2 or CIN3 and positivity for HPV DNA when this group was compared with women with only CIN1 or normal cervix (OR = 7.8; 95% CI 1.5 to 53.4).

**Table 3:** HPV DNA detection and the grade of the lesions in 119 women referred for colposcopy

Hybrid Capture II (1.0 RLU/CO)	Final diagnosis		OR (95% CI)
	Normal Cervix CIN1 (%)	CIN 2 OR 3 (%)	
Negative	62 (59%)	2 (15%)	
Positive	44 (41%)	11 (85%)	7.8 (1.5 to 53.4)
**Total**	**106 (100%)**	**13 (100%)**	

*CIN = cervical intraepithelial neoplasia; OR = odds ratio; CI = confidence interval.*

**Figure 1:**
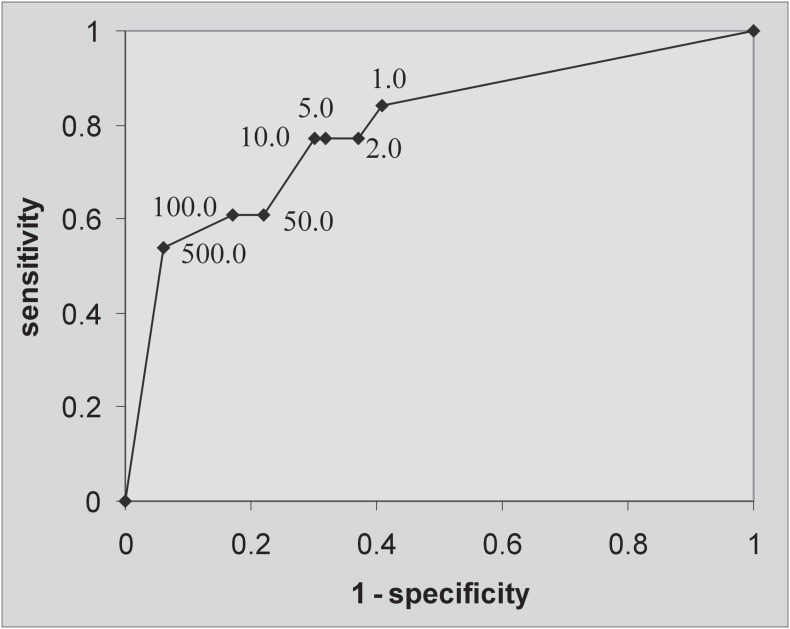
Receiver-operating characteristics (ROC) curve analysis for the performance of the hybrid capture II test in the diagnosis of cervical intraepithelial neoplasia grades 2 or 3 (relative light unit/cutoff, RLU/CO values).

Figure 1 shows the receiver operating characteristic (ROC) curve constructed to describe the sensitivity and specificity of the HCII test at different cutoff points of the DNA index (RLU/CO ratio) for diagnosing CIN2 or CIN3 lesions. The area under the curve is 0.784, thus showing good performance of the HCII in the diagnosis of CIN2 or CIN3. Although the highest sensitivity value in the detection of CIN2 or CIN3 was achieved at the standard manufacturer's recommended point of 1.0 RLU/CO, the best balance in specificity and sensitivity was reached at the cutoff point of 10.0 RLU/CO, showing a 77% sensitivity and 70% specificity. It is important to notice that a slight loss of sensitivity occurred when comparing this point to the standard 1.0 RLU/ CO cutoff, with a concurrent and more pronounced gain in specificity.

The second cervical smear showed ASC or SIL in 13/28 women (46%) with a normal cervix; 70/78 women (89%) with histologically confirmed CIN1 and 11/13 (85%) of those with CIN2 or CIN3. The second cervical test with at least ASC did not accurately differentiate normal cervix or CIN1 from CIN2 or CIN3 (OR = 1.5; 95% CI 0.3 to 10.7) (Table 4).

**Table 4:** Second cervical smear and grade of the lesion in 119 women referred for colposcopy

Second Cervical Smear	Final diagnosis		OR (95% CI)
	Normal Cervix CIN1 (%)	CIN 2 OR 3 (%)	
Normal	23 (22%)	2 (15%)	
ASC/LSIL/HSIL	83 (78%)	11 (85%)	1.5 (0.3 TO 10.7)
**Total**	**106 (100%)**	**13 (100%)**	

*CIN = cervical intraepithelial neoplasia; OR = odds ratio; CI = confidence interval.*

Regarding the Begg-corrected performances of the diagnostic tests, the sensitivities of the second cervical smear and HCII were similar, although the specificity of HCII was significantly higher than second cervical smear (Table 5).

**Table 5:** Begg-corrected performance of the different tests in 119 women referred for colposcopy

	Hybrid capture II (1.0 RLU/CO)	Second cervical smear
Test-positive (total %)	55 (46%)	94 (79%)
True positive	11	11
Proportion of non-verified test-positive (%)	5%	11%
Begg-corrected estimates, % (95% CI)		
Sensitivity	80 (57 - 100)	76 (49 TO 100)
Specificity	59 (48 - 68)	21 (12 TO 29)

*CI= confidence interval.*

## DISCUSSION

Correct identification of women with CIN2 or CIN3 or invasive cancer among those who have been referred because of ASC and LSIL cervical smear results has a major impact on the cost-effectiveness of patient management and triage.^3,4,7,9,11^ To assess the performance of HPV testing as a triage tool, the present cross-sectional study compared repeated cytology, HPV testing (by means of HCII) and colposcopy in a series of women referred to our clinic because of ASC and LSIL cervical smears.

In our series, the detection rate for HPV DNA by HCII was significantly higher in women referred because of LSIL (52%) than in women referred because of ASC (16%). However, in both groups the high-risk HPV detection rate was significantly lower than recently reported from an ongoing ALTS study in the US, in which the high-risk HPV was found in 85% of women with LSIL and in 50% of those with ASCUS.^5,7^ Results closer to ours were reported by Lee et al. (2001), who showed HCII-positive results in 63.8% of women with LSIL and 26.3% of women with ASCUS.^2^ These figures are consistent with the data recently reported from a large-scale screening trial in Russia, Belarus and Latvia (NIS Study), comparing HCII testing with other screening tests.^9^ The results seem to be very similar if the polymerase chain reaction (PCR) is used for HPV detection, as shown by the report of Zerbini et al. (2001), who included HPV types 6, 11, 16, 18, 31, 33, and 45 in their panel and showed 27.3% and 53.5% detection rates in ASCUS and LSIL cases, respectively.^16^

The 11% prevalence of CIN2 or CIN3 in the present series was identical in women who were referred because of ASC and LSIL. Not unexpectedly, the detection of high-risk HPV was significantly associated with the presence of high-grade CIN lesions in the biopsy. Even more importantly, a significant statistical association could be established between the viral load and the high-grade CIN lesions. This is consonant with the data from some recent reports, suggesting that high viral load of HPV16 DNA is a major risk factor for the development of carcinoma in situ (CIS), and measurements of the viral load might be of value in estimating the probability of progressive disease.^17^ The risk of developing CIS, estimated through odds ratios, increased significantly with increasing amounts of HPV16 measured through TaqMan-PCR. Also using PCR, Zerbini et al. (2001) showed that the amount of HPV DNA in the samples varied widely among the women with biopsy-confirmed CIN1, CIN2, CIN3 or invasive cancer.^16^ There was also a major variation in the viral load in women within each single histological category (CIN1, CIN2 or CIN3), as well as between the different groups. These authors attributed the higher viral load in high-grade lesions to HPV16, which was the single most prevalent genotype in these patients. According to these authors, HPV16 load seems to be a specific but not a highly sensitive diagnostic marker for defining cervical disease status.^16^

Even more recently, Abba et al. (2003)^18^ analyzed the relationship between viral type and copy number of HPV DNA with respect to the grade of cervical disease. In this study, the viral load determination was performed using low stringency PCR and these authors classified DNA concentration as 10, 20, 100 and 500 copies of HPV16 per cell, considering 100 viral particles per cell in order to define a cutoff between low and high viral loads. These authors found that, in samples with high-risk HPV, 45% presented high viral load whereas only 18% of samples infected with low-risk virus had a copy number above 100 particles per cell. When only samples harboring HPV16 were considered, almost 50% presented high viral load, which was significantly more than the proportion when considering other high-risk viral types (OR = 2.59; 95% CI = 1.18 to 5.70). In this same study, high-grade lesions were also significantly associated with HPV16 high viral load (OR = 8.53; 95% CI = 2.73 to 26.70).

HC tests are based on a pool of high-risk HPV types and differ from PCR in its linear dynamic range of viral load. Their results are also more influenced by sample cell count. These features make HC results more controversial and difficult to interpret.^19^ Shiffman et al. (2000), showed that women with cytological abnormalities had higher viral load via HCII tests when compared with women with normal cervical smears. They showed that an analytical sensitivity of 1.0 pg/ml using the HCII assay would have permitted detection of 88.4% of high-grade lesions and invasive cancer, whereas the lower levels of detection with HCII (i.e. < 1.0 pg/ml) proved to be clinically non-specific without any gain in diagnostic sensitivity.^20^ These figures are supported by recent data from the NIS study, showing that the presence of high-grade histology was associated with HCII positivity (cutoff of 1 pg/ml) (OR = 4.8; 95% CI = 0.7 to 34.2; p = 0.047). Using this cut-off, the sensitivity of the HCII test was 96.6% (90.0 to 100), specificity 15.9% (10.6 to 21.2), positive predictive value 15.1% (9.9 to 20.3) and negative predictive value (NPV) 96.8% (90.3 to 100) in detecting biopsy-confirmed highgrade lesions.9 Changing the cutoff in either direction did not significantly affect the sensitivity until a sensitivity level of 20.0 pg/ml, and an NPV level of 500.0 pg/ml.

Although the viral load distribution in our series showed a wide range within the histological categories, a viral load of about 10.0 RLU seemed to represent the best-balanced specificity-sensitivity cutoff point in the ROC analysis. The best values for sensitivity and specificity in predicting high-grade lesions were achieved in viral load ranges of between 5.0 RLU/CO and 50.0 RLU/CO, i.e. far above the standard manufacturer's recommendation of a cutoff point of 1.0 RLU/ CO for use in the screening of cervical HPV- related abnormalities.

Sun et al. recently reported that quantitative levels of high-risk HPV DNA were clearly associated with the presence of CIN2 and CIN3 or invasive carcinoma.^21,22^ They believed that increased DNA loads of high-risk HPV DNA, as determined by HCII in cervical specimens, could be used as the specific marker for progressive disease. For practical purposes, however, they considered that the viral load index values (RLU/CO) should be categorized into three groups: low viral loads of < 0.6 RLU/CO, intermediate viral loads of 0.6 to 10.0 RLU/CO and high viral loads of > 10.0 RLU/CO.^21,22^

It is evident, however, that the lowest values (below 1.0 RLU/CO) need to be considered as too low, resulting in a significant increase of false positives, if used as the referral criterion for colposcopy.^9^ Because of these unsolved controversies, there are no generally agreed cutoff values for low, intermediate and high viral loads, as determined by the HCII test (Lorincz, personal communication). However, as far as the correlation with cervical disease is concerned, it now seems that viral loads of less than 1.0 RLU/CO are very low indeed. Sherman et al. (2002) showed that there is a low risk of CIN3 or cancer in the range of 1.0 to 10.0 RLU/CO, and it is reasonable to call these levels low.^5^ Lorincz et al. (2002) suggested a log-range scale definition of viral load, assuming 10.0 to 100.0 to be intermediate levels, 100.0 to 1000.0 high levels and over 1000.0 RLU/CO very high levels.23 Based on experience from the large-scale NIS study, these suggested limits seem feasible and applicable to the majority of cases, although deviations in either direction (i.e. no disease with high RLU/CO values and significant disease with RLU/CO values below 10.0) certainly occur, even in patient material examined for screening purposes.^9^

HPV testing should not be considered only as a diagnostic tool, and determination of HPV types may yield important information regarding prognosis. Some authors have found that the proportion of CIN related to HPV DNA ranged from 90 to 98% and that the maximum risk values were attributed to the presence of HPV16.^24^ The present study has not focused on HPV typing, although it is noteworthy that HPV typing has an important role in the estimation of the risk for high-grade cervical lesions. Moreover, besides viral type, higher viral loads have been implicated in risk augmentation.^25^

## CONCLUSIONS

In the studied population, referred for examination due to an ASC or LSIL cytological smear, HCII testing for high-risk HPV types was a sensitive tool in detecting CIN2 or CIN3, with better diagnostic performance than repeated cervical smears. As the detection rate for high-risk HPV DNA was around 50% in women with LSIL smears (lower than in many other series), the HCII test performed particularly well in this group of women.
